# Bioactivity of the Ethanol Extract of Clove (*Syzygium aromaticum*) as Antitoxin

**DOI:** 10.1155/2023/3245210

**Published:** 2023-09-21

**Authors:** Erwin Afrendi, Muhammad Eka Prastya, Rika Indri Astuti, Wulan Tri Wahyuni, Irmanida Batubara

**Affiliations:** ^1^Department of Biology, Dramaga Campus, IPB University, Bogor 16680, Indonesia; ^2^Research Center for Pharmaceutical Ingredients and Traditional Medicine, National Research and Innovation Agency (BRIN), Kawasan Sains dan Teknologi (KST) B.J Habibie (PUSPIPTEK) Serpong, Tangerang Selatan, Banten 15314, Indonesia; ^3^Tropical Biopharmaca Research Center, Bogor Agricultural University, Taman Kencana Street, IPB Taman Kencana Campus, Bogor 16128, Indonesia; ^4^Department of Chemistry, Dramaga Campus, IPB University, Bogor 16680, Indonesia

## Abstract

Toxic compounds can induce the formation of free radicals (reactive oxygen species (ROS)) which can trigger damage and decrease cell viability. Clove (*Syzygium aromaticum*) contains phenolic compounds that are useful as antioxidants which can reduce ROS toxicity. However, little is known about the antitoxin activity of clove extract. Therefore, this study is aimed at determining the effect of ethanolic clove extract as an antitoxin agent against malachite green (MG) mutagen using the yeast *Saccharomyces cerevisiae* as a model. The methods used to analyze the ability of ethanolic clove extract as antitoxin were decolorization assay and cell viability test towards MG. The phenol contents of leaf and bud extract were 441.28 and 394.73 mg GAE g^−1^ extract, respectively. Clove leaf extract has strong antioxidant activity in vitro (IC_50_ 9.29 ppm for 1,1-diphenyl-2-picrylhydrazyl (DPPH) and 29.57 for 2,2′-azino-bis(3-ethylbenzothiazoline-6-sulfonic acid) (ABTS)). Liquid chromatography quadrupole-mass spectrometry (LC-MS/MS) analysis showed the presence of 4-O-caffeoylquinic acid and several other bioactive compounds, in which these compounds had bioactivity against toxic compound. The addition of extract reduced the ability of *S. cerevisiae* to decolorize malachite green but increased cell viability. Based on the data, clove leaf extract shows the potential antitoxin activity. This research should facilitate a preliminary study to investigate the antitoxin agent derived from cloves leaf extract. Further research to analyze the antitoxin mechanism of this extract in yeast model is interesting to do to provide a comprehensive insight into the potential antitoxin agents of clove leaf extract.

## 1. Introduction

Phytoextract of clove (*Syzygium aromaticum*) is a source of natural ingredients which are reported to have various biological activities that have beneficial effect to human health. Clove is widely used by traditional communities for generations as wound medicine, massage oil, body warmer, and spices for cooking [1]. Clove extract has been reported by some early researchers to have several promising bioactive compounds such as flavonoids, saponins, phenolics, tannins, steroids, terpenoids, and alkaloids [2, 3]. Clove extract has also been studied to have antibacterial and antifungal [4, 5], anti-inflammatory [6], analgesic [7], antioxidant [8], antiglycation, and antiaging [9] activities. However, little is known regarding its activity as antitoxin.

Exposure of toxin, primarily genotoxin agent, towards cellular system may cause various effects including DNA mutations [10]. It is known that DNA mutation is the main cause of genetic diseases. Change in the DNA sequence from its normal sequence may ultimately cause damage in the function of product proteins. High toxicity of toxin has been implied to various metabolic and degenerative diseases such as autoimmune [11], genetic disorder, cancer, and tumors [12]. Various strategies have been carried out to increase cell resistance to genotoxin agents; one of them is the application of antitoxin agents.

Antitoxin agents are expected to increase cell tolerance to compounds that can cause toxicity. Several compounds that have been reported to have antitoxin activity are phenolic compounds. Phenolic compounds have antitoxin activity due to their ability as strong antioxidants. This can be explained because toxic compounds can trigger excess of ROS production in cells, and these ROS are neutralized by the antioxidant activity of phenolic compounds [8]. Some of the high content of phenolic compounds of the Golden Mistletoe Fig (*Ficus deltoidea* var. kunstleri) [13] and the Mascarene Island leaf flower (*Phyllanthus tenellus* Roxb.) [14] were reported to have antigenotoxic activity. Natural resin (propolis) which has high content of phenolic compounds has also been reported to have antitoxin activity, in vitro and in vivo [15]. Based on previous literature information, it shows that clove extract contains high and diverse bioactive compounds, especially phenolic groups with strong antioxidant activity [8]. On the other hand, compounds with high antioxidants were reported as having high antitoxin activity [13], and it is interesting to evaluate the ability of clove extract to reduce toxin (antitoxin agent).

In this study, we used the yeast *Saccharomyces cerevisiae* as a model organism. *S. cerevisiae* has been widely used for studying various cellular phenomena including biotransformation of toxic compounds and cellular impact of toxic substances or materials [16]. As for genotoxin agent, we use malachite green (MG), a chemical dye that has high toxicity and can cause DNA mutations. MG has a toxic effect on mammalian cells by the formation of reactive oxygen species (ROS). MG also acts as a tumor promoter in mammals including humans [17]. Thus, in this study, we primarily analyzed the antioxidant activity and further determine antitoxin potential activity of the ethanolic clove extract in addition to profiling its chemical compounds.

## 2. Materials and Methods

### 2.1. Clove Extract and Yeast Cell Culture

Clove buds and leaves (*Syzygium aromaticum*) were extracted using the maceration method to obtain the ethanol fraction of clove extract [9]. In short, clove buds and leaves were macerated using 70% ethanol at a ratio of 1 : 5 (sample-solvent) in detail of 100 g sample-500 ml solvent, following 24-hour incubation using stirrer. Sample was then collected and concentrated by using the rotary evaporator (45°C) until obtaining clove extract. On the other hand, yeast *Saccharomyces cerevisiae* BY4741 was used as the model organism which was collected from the Laboratory of Microbiology and Bioprospecting, Department of Biology, IPB University, Indonesia. *S. cerevisiae* was routinely maintained in yeast extract peptone dextrose medium (YPD medium). YPD (1 l) was composed of 5 g yeast extract, 20 g glucose, 20 g peptone, 20 g dextrose, and 10 g yeast extract. Agar (20 g/l) was used to make solid medium. Malachite green (MG) 15 ppm was used as toxic compound.

### 2.2. Total Phenolic Content

Determination of the total phenolic content was done with the Folin-Ciocalteu method following the method as described elsewhere [18]. Each of the 10 mg of clove extract (in pellet form) as sample solution was dissolved with 10 ml methanol (concentration of 1 mg/ml) in a volumetric flask size 25 ml. The sample solution was taken for 1 ml and placed into the test tube following the addition with 5 ml of 7.5% Folin-Ciocalteu reagent. The mixture was homogenized with vortex and incubated in a dark room for 8 minutes. After that, NaOH 1% was added as much as 4 ml and rehomogenized and incubated in the dark room for 1 hour. The absorption of the extract solution was read at a wavelength of 730 nm with a UV-Vis spectrophotometer. Gallic acid was used as the standard for determining total phenolic levels in extracts. The phenolic quantity was expressed in GAE (gallic acid equivalent) with the following formula:

(1) $$\begin{array}{c} \text{Total phenolic }\left(\text{GAE}\right)=\frac{\text{concentration of phenolic }\left(\mu \text{g}/\text{ml}\right)\times \text{volume }\left(\text{ml}\right)\;}{\text{mass of extract }\left(\text{mg}\right)}. \end{array}$$

### 2.3. Assessment of Antioxidant Activities

Antioxidant activity of the sample was determined using two types of radicals including 1,1-diphenyl-2-picrylhydrazyl (DPPH) and 2,2′-azino-bis(3-ethylbenzothiazoline-6-sulfonic acid) (ABTS) [18]. Briefly, the sample diluted in methanol in varied concentration including 1000, 500, 250, 125, 61.5, 31.25, 15.125, and 7.5 ppm in each volume of 100 *μ*l was mixed with 100 *μ*l of 125 *μ*M DPPH solution (diluted in methanol) and incubated for 30 minutes prior to being observed for its absorbance. As for ABTS assay, 7 mM ABTS solution mixed with water (H_2_O) was oxidized by 2.45 mM potassium persulfate (diluted on water (H_2_O)) before reacted with the sample with varied concentration like DPPH methods. ABTS radicals were used at the OD_734_ value of 0.68-0.7 by mixing 150 *μ*l of ABTS radicals and 50 *μ*l of each sample concentration following by incubation for 30 minutes. Subsequently, the absorbance of assay was measured by using Thermo Scientific Varioskan Flash (Thermo Fisher) at 515 nm and 734 nm for DPPH and ABTS activity, respectively. The inhibition values were calculated using the formula as follows:

(2) $$\begin{array}{c} \text{DPPH}/\text{ABTS inhibiton value }\left(\%\right)=\frac{{\text{A}}_{0}–{\text{A}}_{\text{S}}}{{\text{A}}_{0}}\times 100\%, \end{array}$$

where *A*_0_ is the absorbance of DPPH/ABTS blank (without samples) and *A*_S_ is the absorbance of samples.

The results are described as inhibitory concentration of 50% (IC_50_). Ascorbic acid and quercetin were applied for the positive control.

### 2.4. Liquid Chromatography Quadrupole-Mass Spectrometry Analysis (LC-MS/MS)

The LC-MS/MS data were obtained by using Waters ACQUITY UPLC I-Class and Xevo G2-XS Qtof following the previous method [19]. Briefly, LC separation was done by using ACQUITY UPLC® BEH C8 1. 7 *μ*m, 2.1 × 100 mm. The mobile phase used was consisted of solvent H_2_O with 0.1% formic acid (FA) and acetonitrile (ACN) with 0.1% formic acid. The mass spectrometer was operated in full scan mode with a scan range of 100–1200 m/z in ESI mode with 1 *μ*l injection volume. Data was processed through UNIFY software program which incorporated it to the instrument. Chromatogram and MS spectrum were analyzed and matched to the corresponding compound using the corresponding software.

### 2.5. Decolorization Assay

Decolorization assay was conducted to see the ability of *S. cerevisiae* in degrading MG after being given clove extract with various concentrations following the method as described elsewhere [8]. Yeast *S. cerevisiae* was grown at room temperature in the yeast extract peptone dextrose broth medium (YPDB) added with clove extract (100, 200, 300, and 400 ppm) total volume of 40 ml. Each medium was added with 15 ppm MG. Yeast culture in YPDB media without the addition of extracts was used as control. All yeast cultures (control and clove extract treatment) were incubated on shaking incubator. The sample was centrifugated at 4000 rpm for 10 minutes. This analysis was conducted by observing its turbidity using a spectrophotometer at the wavelength of 620 nm at an interval of 2 hours for 10 hours. The percentage of decolorization was calculated by the following formula:

(3) $$\begin{array}{c} \%\text{decolorization}=\frac{\text{initial absorbance}-\text{observed absorbance}}{\text{initial absobance}}\times 100\%. \end{array}$$

### 2.6. Cell Viability Assay

Yeast viability was analyzed following MG exposure which refers to the previous method [9]. Yeast culture and MG treatments were prepared as previously described in decolorization assay. The concentrations of yeast cells were quantified at 0 and 10 hours following MG exposure. A 100 *μ*l of each culture was serially diluted in 9.9 ml of 0.85% saline solutions (up to 10^−5^). Each dilution solution was then spread into a Petri dish containing yeast extract peptone agar (YPDA). The Petri dish was incubated at 30°C for 3 days. The number of colonies formed at the 0 and the 10 hours was counted.

### 2.7. Statistical Analysis

All experiments were carried out in triplicate. Data were expressed as mean ± standard deviations. Further analysis was conducted with one-way ANOVA followed by multiple Duncan test range. A *P* value of <0.05 was considered as significantly different.

## 3. Results

### 3.1. Total Phenolic Content

The phenolic content of clove leaves was higher than clove buds (Table 1). The total phenolic content of clove leaves was 441.28 mg GAE g^−1^ extract, while clove bud extract was 394.73 mg GAE g^−1^ extract. Therefore, clove leaf extract was used in the next method.

### 3.2. Antioxidant Activity of Ethanol Clove Leaf Extract

The scavenging effects of ethanol clove leaf extract on the DPPH and ABTS radicals were expressed as IC_50_ value, in which lower IC_50_ indicates higher antioxidant activity. Based on the results obtained, the ethanolic clove leaf extract has an IC_50_ value of 9.29 ± 0.78 and 29.57 ± 0.91 ppm for DPPH and ABTS radicals, respectively. These values were close to ascorbic acid as positive control, with an IC_50_ value that is twice than ascorbic acid, for both DPPH and ABTS radicals (Table 2). These results indicated that this extract has strong antioxidant activity and potentially can be used for further analysis.

### 3.3. Chemical Profile of Ethanol Clove Leaf Extract (LC-MS/MS)

**Figure 1:**
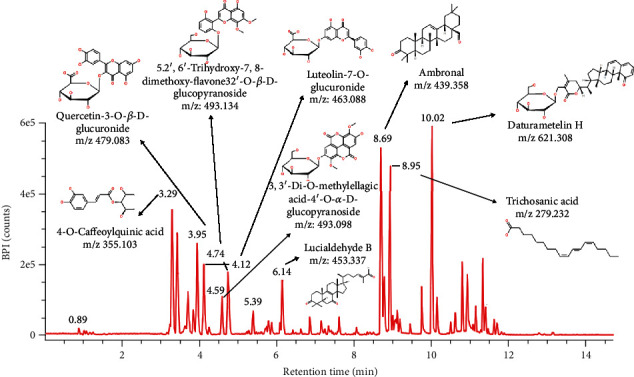
LC-MS/MS profile and predicted compounds of the ethanol clove leaf extract.

Results from LC-MS/MS analysis indicated that the clove leaf extract has more than 10 predicted compounds as shown in Figure 1.

LC-MS/MS analysis showed the presence of 4-O-caffeoylquinic acid, ambronal, daturametelin H, quercetin-3-O-*β*-D-glucuronide, trichosanic acid, and candidate mass C_35_H_36_N_4_O_5_ which was thought to be included in the pheophorbide compound. These compounds had indeed been reported to have several activities against toxic compounds (Table 3).

### 3.4. Decolorization Test

**Figure 2:**
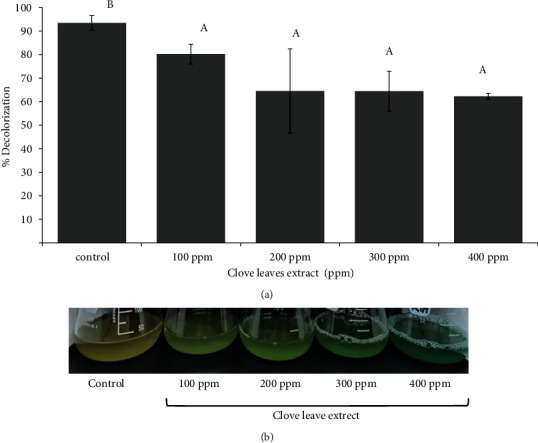
Effect of clove leaf extract on (a) the decolorization percentage of toxic compound malachite green (15 ppm) by *S. cerevisiae* after incubation for 10 hours. (b) Yeast culture appearance following MG treatment after 10 hours of incubation. Culture of *S. cerevisiae* without clove leaf extract was used as control. 15 ppm MG was added on each culture as toxic compound treatments. The same superscript letter in Figure 1(a) representing not significantly different by one-way ANOVA followed by multiple Duncan test range (^A,B^*P* < 0.05).

The results of the decolorization assay showed a decrease in the percentage of MG decolorization by *S. cerevisiae* with the addition of clove leaf extract concentration (Figure 2). The highest and lowest percentages of decolorization were the treatment without the addition of clove leaf extract (control) with a value of 93.48% and the addition of 400 ppm extract with a value of 62.26%, respectively.

### 3.5. Cell Viability Assay

**Figure 3:**
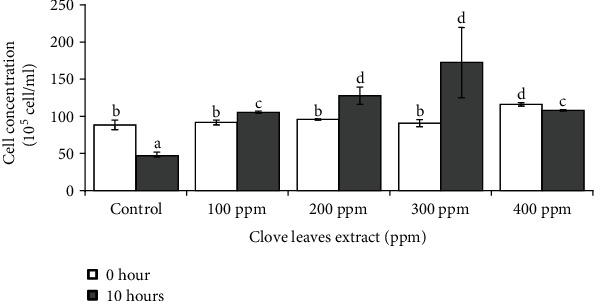
Effect of clove leaf extract on the viability of MG-treated *S. cerevisiae* cells after 10 hours of incubation. Cultures of *S. cerevisiae* that were not given clove leaf extract were used as control. Culture of *S. cerevisiae* without clove leaf extract was used as control. 15 ppm MG was added on each culture as toxic compound treatments. The same superscript letter in Figure 1(a) representing not significantly different by one-way ANOVA followed by multiple Duncan test range (^a-d^*P* < 0.05).

Cell viability assay showed a decrease in the concentration of cells in the control (without the addition of clove leaf extract) (Figure 3). This was because the addition of MG caused oxidative stress that leads to cell death. The addition of clove extract with concentrations of 100 ppm to 300 ppm showed an increase in cell viability as indicated by an increased in the number of colonies. This indicates that the ethanol extract of clove leaves has an antitoxin effect that combat the toxicity of MG. However, slight decrease on the cell viability was found in 400 ppm clove leaf extract treatment. It was likely attributed with the osmolarity that was developed with high concentration of the clove extract treatment on yeast culture. With a high level of osmolarity in the treatment medium due to 400 ppm clove leaf extract treatment, the yeast cells will be damaged because the water component of the cells will be forced out to stabilize with environmental conditions [36]. As a result, many cells die, and their viability decreases as shown in Figure 3.

## 4. Discussion

Phenolic compounds are the most widespread secondary metabolites in the plant kingdom (plantae) and have natural antioxidant potential. Total phenolic compounds of clove extract were found higher than that of bud extract (Table 1). It is highly correlated to the high rate of photosynthesis on leaves, thus providing more abundant biosynthetic precursors for phenolic compound synthesis [37]. Such higher content of phenolic compounds of that clove leaf extract was attributed with strong antioxidant activity based on the in vitro assay (DPPH and ABTS assays) (Table 2). This result is in line with several other studies which showed that high total phenolic content derived from plant extracts, *Castanea sativa* Mill. and *Commiphora mollis* (Oliv.) Engl., has implications for strong antioxidant activity using the same test method, namely, DPPH and ABTS radicals [38, 39]. With this high antioxidant activity, clove leaf extract also has suggested as having good antitoxin ability through the free radical scavenging mechanism found in toxin compounds. In addition, it is worth noting that the ethanol extraction method was suitable in isolating those chemical compounds which elicit antioxidant activity. Several other studies have also used the same ethanol solvent to extract bioactive compounds from some vegetables which have been shown to have strong antioxidant activity [40]. Furthermore, due to it having a strong antioxidant activity, clove leaf extract was chosen for further analysis of its antitoxin ability, starting with an analysis of the profile of the compounds contained in it using LC-MS/MS.

Analysis with LC-MS/MS showed several bioactive compounds contained in the ethanol leaf extract of clove. These compounds show some activities against toxic compounds. Several compounds that have antioxidant activity such as 4-O-caffeoylquinic acid [20], quercetin-3-O-*β*-D-glucuronide [25], trichosanic acid [27], 3,3′-di-O-methylellagic acid-4′-O-*α*-D-glucopyranoside [29], lucialdehyde B [32], 5,2′,6′-trihydroxy-7,8-dimethoxy-flavone-2′-O-*β*-D-glucopyranoside [31], and candidate mass C_35_H_36_N_4_O_5_ (pheophorbide compound) [35] are important to fight oxidative stress caused by toxic compounds. The presence of these potential compounds indicates that the ethanol clove leaf extract is thought to have a synergistic effect to neutralize toxic compounds, so it has the potential to be developed as an antitoxin agent.

In this study, we found that clove leaf extract elicited a strong antioxidant activity both towards DPPH and ABTS radicals. However, relatively high concentration of extract (about >5 times IC_50_ or 100-300 ppm) has no negative effects towards yeast cells indicating that their cell viability is still high as previously reported [9]. This data indicates that clove leaf extract may not be acted as prooxidant intracellularly. This can be seen from the value of the antioxidant activity obtained which is classified as strong antioxidant activity, so the activity of clove leaf extract tends to be through an antioxidant mechanism. This mechanism can occur by reducing free radicals in the environment because at high concentrations, they have no effect on cells as described above [9]. However, a considered very high clove leaf extract treatment (400 ppm) caused a slight decrease on yeast cell survival rate following MG exposure. Cytotoxicity of MG has been reported due to development of ROS leading to oxidative stress conditions [41]. Thus, application of such strong antioxidant properties of clove leaf extract is a potential strategy to combat ROS-mediated MG toxicity.

The toxic substance of MG is due to the formation of triarylmethane dye in the form of crystalline solids which can cause mutations in DNA. MG is commonly used to control the pathogenic fungus in water environment and used as a direct dye for wool, silk, hemp, and leather [17]. MG has also been reported to ultimately compromise cellular activities mediated by beta-arrestin in human which is important for proliferation and apoptosis signaling [42]. As in model yeast, MG causes significant extend on the lag phases. *S. cerevisiae* commonly used for model organisms for studying some cellular mechanisms including nutrient sensing signaling, redox homeostasis, aging, autophagy, and cellular effect of toxic substances [43]. Our data showed that addition of clove extract could promote cell viability of the model yeast *S. cerevisiae*, yet reducing its corresponding decolorization activity towards MG. *S. cerevisiae* has the ability to decolorize textile dyes including MG. MG degradation process involves the induction of several enzymes, namely, laccase, lignin peroxidase, nicotinamide adenine dinucleotide-dichlorophenolindophenol (NADH-DCIP) reductase, aminopyrine N-demethylase, and MG reductase [44]. Such reduction on the decolorization activity of clove extract-treated yeast cells was likely due to physiological switch to activate oxidative stress response caused by MG-mediated ROS production rather than activating the dye-degrading enzyme activity [45]. Such phenomenon has been reported previously which showed that the addition of phytoextracts of *Terminalia chebula*, *Clitoria ternatea*, and *Boerhavia diffusa* which had antioxidant effects could promote antitoxin activity towards MG [41]. In this regards, reduction on the decolorization activity was recorded simultaneously with the increase of cell viability of yeast.

To the best of our knowledge, this is the first report of potential antitoxin activity of the ethanol extract of clove. Results of this study indicate that clove leaf extract has antitoxin activity which was analyzed on the yeast model organism *S. cerevisiae*. Further study on the DNA repair activity of this extract is required to reveal its mode of action as an antigenotoxic agent.

## 5. Conclusions

Clove leaf extract with a total phenolic content of 441.28 mg GAE/g extract and antioxidant activity against DPPH and ABTS radicals (IC_50_ value of 9.29 and 29.57 ppm) and several bioactive compounds has the ability as an antitoxin agent against toxic compound malachite green (MG). Indeed, the addition of clove extract concentrations (200 and 300 ppm) could increase yeast cell viability following MG exposure. These results indicate that clove leaf extract has strong antioxidant activity in line with antitoxin activity. This research is the preliminary process in developing the potential of this extract as an antitoxin agent. Further research is needed to analyze the antitoxin mechanism of the extract against MG. In addition, an analysis of the antitoxin mechanism in the yeast model *S. cerevisiae* also needs to be carried out as a comprehensive in vitro study to reveal the antitoxin potential of clove leaf extract. Ultimately, this research should facilitate the development of antitoxin agents derived from phytoextracts that could in the future be applied to functions such as combating cellular toxic agents or inducing cellular tolerance to toxic compounds.

## Figures and Tables

**Table 1:** Total phenolic from leaf and bud extract of clove.

Sample	Phenolic content (mg GAE/g extract)
Clove leaves	441.28^b^
Clove buds	394.73^a^

Note: the different superscript letters in the same column represent significantly different phenolic content by one-way ANOVA followed by multiple Duncan test range (^a,b^*P* < 0.05).

**Table 2:** Antioxidant activity of ethanol clove leaf extract.

Sample	IC_50_ of DPPH (ppm)	IC_50_ of ABTS (ppm)
Clove leaves	9.29 ± 0.78^c^	29.57 ± 0.91^c^
Ascorbic acid (+)	4.71 ± 0.69^b^	18.35 ± 0.82^b^
Quercetin (+)	2.61 ± 0.86^a^	8.61 ± 0.73^a^

Note: the same superscript letter in the same column representing not significantly different by one-way ANOVA followed by multiple Duncan test range (^a-c^*P* < 0.05).

**Table 3:** LC-MS compound profile of ethanol clove leaf extract.

Component name	Analysis	Formula	Activity
4-O-Caffeoylquinic acid	m/z: 355.1034 RT (min): 3.30	C_16_H_18_O_9_	Antioxidant [20]

Ambronal	m/z: 439.3580 RT (min): 8.69	C_30_H_46_O_2_	Proapoptosis, anticancer [21]

Daturametelin H	m/z: 621.3084 RT (min): 10.02	C_34_H_46_O_9_	Anticancer [22]

Quercetin-3-O-*β*-D-glucuronide	m/z: 479.0827 RT (min): 3.95	C_21_H_18_O_13_	Antiaging [23], anticancer [24], antioxidant [25]

Trichosanic acid	m/z: 279.2324 RT (min): 8.89	C_18_H_30_O_2_	Anticancer and proapoptosis [26], antioxidant [27], and protection against sodium arsenite (SA) toxicity [28]

3,3′-Di-O-methylellagic acid-4′-O-*α*-D-glucopyranoside	m/z: 493.0981 RT (min): 4.59	C_22_H_20_O_13_	Antioxidant [29] and anti-inflammatory [30]

5,2′,6′-Trihydroxy-7,8-dimethoxy-flavone-2′-O-*β*-D-glucopyranoside	m/z: 493.1340 RT (min): 4.76	C_23_H_24_O_12_	Antioxidant [31]

Lucialdehyde B	m/z: 453.3376 RT (min): 6.14	C_30_H_44_O_3_	Anticancer, antioxidant, and antiaging [32]

Luteolin-7-O-glucuronide	m/z: 463.0880 RT (min): 4.12	C_21_H_18_O_12_	Antigenotoxin, antimutagenic, antioxidant, proapoptosis [33]

Candidate mass C_35_H_36_N_4_O_5_	m/z: 593.2774 RT (min): 8.94	C_35_H_36_N_4_O_5_	Proapoptosis, anti-inflammatory [34], antioxidant [35]

Candidate mass C_48_H_82_N_2_O_17_	m/z: 959.5702 RT (min): 10.81	C_48_H_82_N_2_O_17_	No data

## Data Availability

The data used to support this study are provided within the article.
